# A Case of Membranous Croup

**Published:** 1853-09

**Authors:** S. H. Bristol

**Affiliations:** Parma, Michigan


ART. II.—*A case of Membranous Croup, with the discharge of a Tube of False Membrane from the Bifurcation of the Trachea.* By Dr. S. H. Bristol, Parma, Michigan.

On the 28th of December, I was called to see a son of Mr. H., aged 6 years. Found him laboring under Pseudo Membranous Croup, pulse 100 and small, breathing very laborious, face and lips livid, countenance pale and haggard, gave him an emetic; he threw up a considerable quantity of glary mucous, and some shreds of false membrane. After vomiting was over, was much relieved, pulse 90, full and soft, face and lips natural. Ordered full dose of calomel to act on the bowels; after which gave ant. gr. 1-6, opi. ¼, to be given every 3 hours.

December 29, found him much improved from previous night; no traces of disease left, except a slight hoarseness in coughing. Ordered hive syrup to be given; and left, with strict orders not to let him be exposed to the cold air.

Jan. 2, three days after, was called in great haste, found his breathing very laborious, pulse 120 and small, face livid; vomited him freely, he threw up several pieces of pseudo membranous exuadtion. Ordered calomel gr. 2, opi. $\frac{1}{4}$, ipecac. $\frac{1}{2}$, every four hours; alternated with stimulating expectorants. Remained easy until 6 o'clock, P. M., when the breathing became laborious; gave ant. $\frac{1}{4}$ gr. every fifteen minutes until free emesis took place; threw up, at this time, an entire tube, from the bifurcation of the trachea; the part extending up the trachea, from the bifurcation, was one inch in length; from the bifurcation, to the right lung, one inch and one-fourth; to the left lung, three-fourths of an inch; making the entire length of the tube, on the right side, two and one fourth inches in length. Also a large quantity of thick, tenacious mucous.

Jan. 3, remained easy for several hours, when dyspnœa, livid features, jactation, and other unfavorable symptoms came on, which resulted in death, on the third day of the second attack. The peculiarities in this case, seemed to be, the entire tube, its length, the amount of plastic exudation, and the rapidity with which it was formed, after being discharged.

